# A Real-Time SDR-Based Vehicular Scatterometer with Multi-Subband Coherent Synthesis

**DOI:** 10.3390/s26092891

**Published:** 2026-05-05

**Authors:** Shijie Yang, Wei Guo, Caiyun Wang, Peng Liu, Te Wang, Zhenzhen Liang, Qing Xing, Xingming Zheng, Bingze Li

**Affiliations:** 1 The CAS Key Laboratory of Microwave and Remote Sensing Technology, National Space Science Center, Chinese Academy of Sciences, Beijing 100190, China; yangshijie21@mails.ucas.ac.cn (S.Y.); wangcaiyun@mirslab.cn (C.W.); liupeng@mirslab.cn (P.L.); wangte@mirslab.cn (T.W.); liangzhenzhen211@mails.ucas.ac.cn (Z.L.); xingqing23@mails.ucas.ac.cn (Q.X.); 2 University of the Chinese Academy of Sciences, Beijing 100049, China; 3 State Key Laboratory of Black Soils Conservation and Utilization, Northeast Institute of Geography and Agroecology, Chinese Academy of Sciences, Changchun 130102, China; zhengxingming@iga.ac.cn (X.Z.); libingze@iga.ac.cn (B.L.); 4 School of Biological and Agricultural Engineering, Jilin University, Changchun 130022, China

**Keywords:** software-defined radio, ground-based scatterometer, stepped-frequency, subband synthesis, pulse compression, full-polarization

## Abstract

Ground-based scatterometers are widely used for quantitative microwave backscattering measurements in soil moisture retrieval, vegetation monitoring, and satellite scatterometer validation. However, low-cost software-defined radio (SDR) transceivers provide limited instantaneous bandwidth, making it difficult to transmit and process signals with bandwidths on the order of hundreds of MHz for fine range resolution, especially for systems requiring real-time onboard processing. To address this problem, this paper presents a vehicular, fully polarimetric, SDR-based scatterometer that achieves an equivalent wideband response by sequentially transmitting adjacent narrow subbands and coherently synthesizing them onboard. To enable real-time operation on a resource-limited field-programmable gate array/system-on-chip (FPGA/SoC) platform, we adopt a frequency-domain synthesis-pulse-compression pipeline that avoids interpolation and eliminates repeated matched filtering across subbands. A slot-based online phase calibration is performed within the settling window after each fast lock to estimate and compensate random local oscillator (LO) phase offsets, preserving coherent stitching. In addition, pulse repetition within each subband and coherent accumulation are integrated to improve the signal-to-noise ratio (SNR) under real-time throughput constraints. A Zynq-based implementation demonstrates deterministic onboard range-profile output, with a minimum processing latency of about 1.57 ms per frame. Loopback and outdoor experiments validate the equivalent 200 MHz bandwidth (five 40 MHz subbands), achieving approximately 0.75 m resolution and yielding sidelobe metrics consistent with the designed windowing, including a peak sidelobe ratio (PSLR) of −27.43 dB and an integrated sidelobe ratio (ISLR) of −12.38 dB. Field scans over farmland further show consistent $\sigma_{0}$ trends across incidence angle and azimuth, indicating reliable onboard quantitative backscattering measurement. These results demonstrate that the proposed method provides a feasible solution for deterministic real-time equivalent wideband scatterometry on a low-cost SDR platform.

## 1. Introduction

Ground-based scatterometers are widely used for quantitative microwave backscatter measurements in soil-moisture retrieval, vegetation monitoring, and ground validation of satellite scatterometers [1,2,3,4]. In recent years, related studies on sensing systems for complex environments and resource-constrained platforms have generally shown that system performance depends not only on the algorithm itself, but also on the combined effects of bandwidth constraints, data throughput, hardware resources, and real-time requirements [5,6,7]. In microwave sensing scenarios, this issue is equally prominent: the system must provide sufficient equivalent bandwidth to achieve fine range resolution, while also maintaining stable and repeatable real-time operation on low-cost, reconfigurable, and compact platforms. To obtain fine range resolution, the system typically needs to transmit and process wideband linear frequency-modulated (chirp) signals with bandwidths on the order of hundreds of MHz. However, the instantaneous bandwidth of low-cost SDR radio frequency (RF) transceivers is limited, making a directly wideband design difficult in practice. For example, some recent GNU Radio-based continuous-wave radar implementations were still constrained by limited bandwidth and therefore did not provide high range resolution; similarly [8], one pseudo-random-noise radar reported a maximum tunable bandwidth of only 16.384 MHz and explicitly suggested that broader-band modulation would be needed to further improve range resolution [9]. Therefore, this paper adopts a stepped-frequency (multi-subband) scheme [10]: multiple relative narrow subband signals in adjacent frequency ranges are transmitted sequentially, and the received echoes are coherently synthesized across subbands to form an equivalent wideband echo, on which real-time pulse compression is performed to output a range profile. It should be noted that many SDR-based stepped-frequency systems still rely on offline host processing [11,12,13], incurring large data-transfer and end-to-end latency costs and making closed-loop real-time feedback in field experiments difficult. Accordingly, this work targets onboard real-time processing for an SDR scatterometer with limited instantaneous bandwidth, aiming to achieve deterministic and repeatable real-time output on a resource-constrained FPGA/SoC platform [14].

Regarding the implementation domain for the “synthesis–pulse compression” chain, existing methods can be summarized along two dimensions: (1) the domain (time-domain synthesis or frequency-domain synthesis) and (2) the processing order (synthesize first, then compress, or vice versa). Time-domain synthesis [15] usually dechirps each subband echo first and then constructs an equivalent wideband signal by time shifting and concatenation; this process often relies on interpolation and frequency shifting, leading to high computational and implementation complexity. Moreover, amplitude/phase inconsistencies at subband boundaries are more likely to appear as time-domain discontinuities, which degrades sidelobe control after pulse compression. Based on these considerations, this paper chooses a frequency-domain synthesis route: subbands are aligned by spectral shifting and stitching, avoiding interpolation; the structure is more regular, processing is faster, and it is convenient for streaming FPGA pipelines, which is more suitable for real-time systems.

Within the frequency-domain route, prior work often follows the order of “pulse compression first, then synthesis”. Yang Hui [16] proposed performing matched filtering on each subband to obtain subband-compressed results, followed by alignment, phase compensation, and synthesis; subsequent studies [17,18,19,20] further improved delay errors and phase discontinuities. However, such methods typically depend on offline processing on the processing system (PS) side or a PC [21]: on the one hand, it’s not well suited for online real-time phase calibration and typically performs phase correction through offline post-processing of stored echoes and reference signals; on the other hand, pulse compression must be repeated for each subband and more intermediate results must be stored, so the computation and data-movement overhead grows with the number of subbands N, leading to large end-to-end latency and throughput pressure, which is unfavorable for online real-time output. In contrast, Lord et al. [22] proposed the order of “frequency-domain alignment/stitching first, then wideband matched filtering”: each subband spectrum is shifted onto a common frequency axis and stitched to form an equivalent wideband spectrum, followed by a single wideband matched filter for pulse compression. This order is structurally more amenable to real-time implementation, but the synthesis result is highly sensitive to inter-subband phase consistency: after each frequency fast lock, the SDR transmit/receive (TX/RX) local oscillators may introduce mismatches such as random initial phase [23,24]; if not suppressed, the stitching coherence degrades and pulse-compression sidelobes rise. Meanwhile, the increased data volume due to multiple subbands further squeezes on-chip memory bandwidth and computation budget. Therefore, how to simultaneously balance equivalent-bandwidth construction, inter-subband phase-consistency maintenance, and onboard real-time processing efficiency on a low-cost SDR platform remains a key problem in current engineering implementation.

To address the above issues, this paper focuses on the onboard real-time implementation of a low-cost vehicular SDR-based scatterometer and mainly carries out the following three tasks: (i) a pipeline that performs frequency-domain synthesis before pulse compression, thereby reducing repeated computations and intermediate buffering; (ii) an interleaved online phase-calibration method that estimates subband phase within the fast-frequency-switching settling window and compensates it in real time; (iii) a within-subband pulse repetition and coherent accumulation strategy that improves SNR while maintaining real-time throughput. Together, these methods constitute a real-time multi-subband coherent-synthesis processing architecture for resource-constrained FPGA/SoC platforms, providing a feasible solution for realizing equivalent-wideband measurements with a low-cost SDR-based ground scatterometer.

## 2. Principles and Methods

### 2.1. Principles

#### 2.1.1. Multi-Subband Chirp

Let the wideband transmitted signal be a chirp with center frequency $f_{c}$, total bandwidth B, chirp rate k, and pulse width $T_{p}$, expressed as:

(1) $$s(t)=Arect\left(T_{p}\right)\exp\left(j\pi kt^{2}\right)\exp\left(j2\pi f_{c}t\right)$$

where A is an amplitude constant and rect(·)  denotes a rectangular window.

Limited by the instantaneous bandwidth of the SDR, a stepped-frequency multi-subband waveform is adopted: the equivalent total bandwidth B is divided into N adjacent subbands, each having an identical effective bandwidth $B_{N}=B/N$.

**Figure 1:**
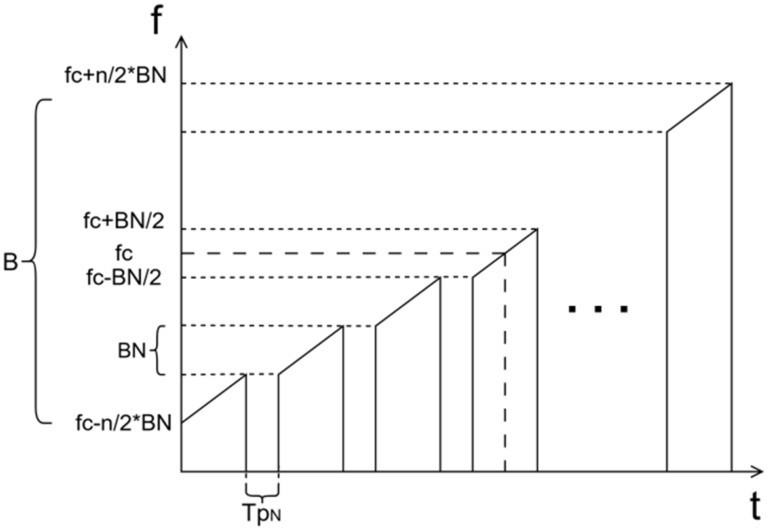
Illustration of a multi-subband stepped-frequency waveform.

The center frequency $f_{c}(i)$ of the i-th subband is:

(2) $$f_{c}(i)=f_{c}+\left(i-\frac{1}{2}-\frac{n}{2}\right)B_{N}$$

where i=1,2,3,…,N, the chirp rate of each subband remains k, and the pulse width is $T_{pN}=T_{p}/N$. The i-th transmitted signal of the stepped chirp can be written as:

(3) $$s_{i}(t)=A_{i}rect\left(\frac{t}{T_{pN}}\right)\exp\left(j\pi kt^{2}\right)\exp\left(j2\pi f_{c}(i)t\right)$$

where $A_{i}$ denotes the signal amplitude of the i-th transmitted subband. The partitioning is illustrated in Figure 1.

Assuming the target is stationary at $R_{0}$, the target echo is:

(4) $$r_{i}(t)=B_{i}rect\left(\frac{t-\tau_{0}}{T_{pN}}\right)\exp\left[j\pi k({t-\tau_{0})}^{2}\right]\exp\left[j2\pi f_{c}(i)(t-\tau_{0}\right)]$$

where $B_{i}$ denotes the received-signal amplitude of the i-th subband, $\tau_{0}=2R_{0}/c$ denotes the target time delay, and c is the speed of light.

Ideally, the complex baseband signal after quadrature demodulation at the receiver is:

(5) $$z_{i}(t)=C_{i}rect\left(\frac{t-\tau_{0}}{T_{pN}}\right)\exp\left[j\pi k({t-\tau_{0})}^{2}\right]\exp(-j2\pi f_{c}(i)\tau_{0})$$

where $C_{i}$ denotes the baseband amplitude of the demodulated i-th subband.

In practical measurements, the transmit and receive local oscillators of a low-cost SDR are usually non-coherent, which introduces a significant system phase error. Therefore, the measured complex baseband signal is:

(6) $$z_{i}(t)=C_{i}rect\left(\frac{t-\tau_{0}}{T_{pN}}\right)\exp\left[j\pi k({t-\tau_{0})}^{2}\right]\exp[-j2\pi {(f}_{c}(i)\tau_{0}-\phi_{i})]$$

where $\phi_{i}=\phi_{t,i}-\phi_{r,i}$, $\phi_{t,i}$ denotes the initial phase of the transmit LO for the i-th signal, and $\phi_{r,i}$ denotes the initial phase of the receive LO for the i-th signal.

#### 2.1.2. Frequency-Domain Synthesis Processing

**Figure 2:**
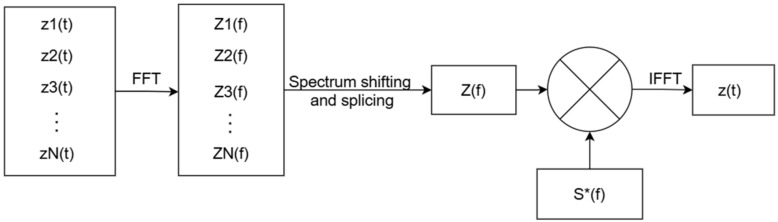
Signal-processing flowchart.

To fit real-time streaming implementation on FPGA, this paper adopts an online processing order of “frequency-domain synthesis first, then unified pulse compression”, as shown in Figure 2.

First, zero-padded fast Fourier transform (FFT) is applied to the phase-corrected subband echoes to improve frequency-axis alignment accuracy. Let the frequency-domain representation of the zero-padded FFT for the i-th subband be:

(7) $$Z_{i}(f)=D_{i}rect\left(\frac{f}{B_{i}}\right)\exp\left[-j\pi \frac{f^{2}}{k}\right]\exp\left[-j2\pi {(f\tau_{0}+f}_{c}(i)\tau_{0}-\phi_{i}\right)]$$

where $D_{i}$ denotes the spectral magnitude of the i-th subband.

To stitch subbands, each subband spectrum is shifted to a common synthesis frequency axis. The frequency shift for the i-th subband is defined as:

(8) $$∆f_{i}=\left(i-\frac{1}{2}-\frac{n}{2}\right)B_{n}$$

After shifting, the spectrum of the i-th subband becomes:

(9) $$Z_{i}(f)=D_{i}rect\left(\frac{f-∆f_{i}}{B_{i}}\right)\exp\left[-j\pi \frac{({f-∆f_{i})}^{2}}{k}\right]\exp[-j2\pi {(f-∆f_{i}+f}_{c}(i){)\tau }_{0}+j2\pi \phi_{i}]$$

**Figure 3:**
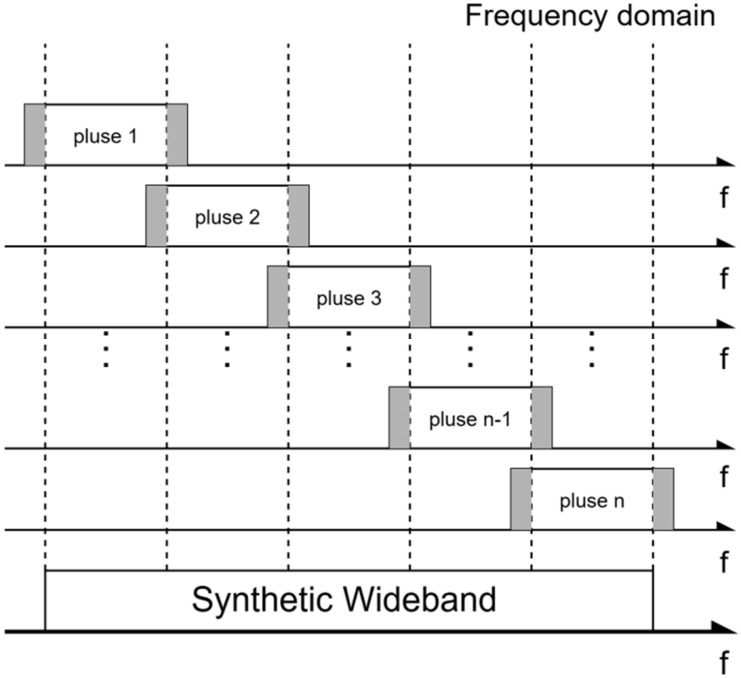
Schematic of multi-subband spectral stitching to form a synthesized wideband spectrum.

Considering that adjacent subbands may overlap at the edges, the overlapped bands are removed before stitching to avoid spectral ripples caused by coherent superposition in the overlap region. Figure 3 shows the process of removing overlaps and stitching subbands in the frequency domain to form a synthesized wideband spectrum. After stitching, the synthesized spectrum is obtained as:

(10) $$Z(f)=rect\left(\frac{f}{B}\right)exp(-j2\pi f\tau_{0})exp(-j2\pi \frac{f^{2}}{k})\exp(-j2\pi {f_{c}\tau }_{0})U(f)$$

where U(f) describes the equivalent complex weighting introduced by subband stitching, written as:

(11) $$U(f)=\sum_{n=1}^{N}D_{i}rect\left(\frac{f-∆f_{i}}{B_{i}}\right)\exp\left(-j\pi \frac{{2f∆f_{i}+∆f_{i}}^{2}}{k}+j2{\pi \phi }_{i}\right)$$

#### 2.1.3. Unified Matched Filtering and IFFT-Based Pulse Compression

To ensure consistent matching for the “synthesize first, then compress” chain, the reference signal undergoes the same zero-padded FFT, frequency shifting, and stitching to obtain the synthesized reference spectrum S(f) (corresponding to the time-domain signal s(t)), based on which the matched filter $S^{*}(f)$ (corresponding to the time-domain signal $s^{*}(-t)$) is constructed. For pulse compression, frequency-domain multiplication is used instead of time-domain convolution. By the convolution theorem, time-domain convolution is equivalent to frequency-domain multiplication, i.e.:

(12) $$Z_{0}(f)=F\{z_{0}(t)\}=F\{z(t)⨂s^{*}(-t)\}=F\{z(t)\}\cdot F\{s^{*}(-t)\}=Z(f)S^{*}(f)$$

where ⨂ denotes linear convolution. Compared with performing an L-length convolution directly in the time domain (computational complexity about $O(L^{2})$), the frequency-domain implementation has overall complexity about O(LlogL). Therefore, when the effective number of points to be processed after multi-subband synthesis is large, frequency-domain multiplication can significantly reduce online computation and end-to-end latency, which is beneficial for real-time echo processing.

Under ideal matching, the frequency-domain pulse-compression result $Z_{0}(f)$ of the synthesized signal can be written as:

(13) $$Z_{0}(f)=Erect\left(\frac{f}{B}\right)exp[-j2\pi (f{+f_{c})\tau }_{0}]\sum_{n=1}^{N}D_{i}rect\left(\frac{f-∆f_{i}}{B_{i}}\right)exp(j2{\pi \phi }_{i})$$

where E denotes the amplitude coefficient of the pulse-compression result in the frequency domain.

Applying inverse fast Fourier transform (IFFT) to yields the time-domain compressed waveform of the synthesized wideband signal:

(14) $$z_{0}(t)=Fsinc[B(t-\tau_{0})]exp({-j2\pi f}_{c}\tau_{0})\omega (t)$$

where F denotes the amplitude coefficient of the pulse-compression result in the time domain, and the weighting function ω(t) is an equivalent weighting caused by inter-subband amplitude/phase inconsistency:

(15) $$\omega (t)=F^{-1}\{\sum_{n=1}^{N}D_{i}rect\left(\frac{f-∆f_{i}}{B_{i}}\right)exp(j2{\pi \phi }_{i})\}$$

The range resolution of the synthesized wideband signal is:

(16) $$\rho =\frac{c}{2B}=\frac{c}{2nB_{n}}$$

It can be seen that subband stitching significantly increases the equivalent bandwidth and thus improves range resolution.

### 2.2. Methods

#### 2.2.1. Time-Interleaving Method

**Figure 4:**
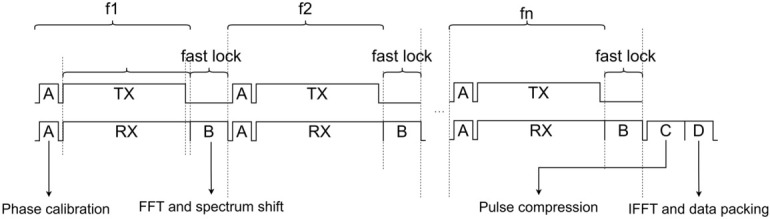
System-level timing diagram. A is phase calibration phase; B is FFT and spectrum shift phase; C is pulse compression stage; D is the IFFT and data packing phase; TX and RX represent the transmit and receive phases, respectively.

As shown in (14), the random LO phase $\phi_{i}$ of the SDR directly affects the mainlobe and sidelobe shapes: In the presence of phase errors or other non-coherent errors, the pulse compression result of the synthesized wideband signal is likely to suffer from mainlobe broadening, energy dispersion, and increased sidelobe levels, which in turn reduce the accuracy of range estimation and degrade the range resolution; coherent stitching is highly sensitive to inter-subband phase. Therefore, the system performs online calibration after each frequency fast lock. The overall timing is organized as a main loop of “fast lock–calibrate–measure–process–output” (Figure 4), and a time-interleaving method is proposed.

In the time-interleaving method, within each subband period, a calibration slot is placed between “fast lock completed” and “measurement window start” to quickly perform a loopback measurement under the same frequency configuration and write the phase-compensation term $\phi_{i}$ for that subband; the system then enters the measurement window to acquire echoes. To improve real-time utilization, the digital processing of the previous subband (FFT/spectral shifting, etc.) is overlapped in parallel with the RF fast lock reconfiguration of the next subband, avoiding serial waiting. The critical path per frame can be summarized as “measurement window + max (fast lock configuration time, digital processing time) + fixed pulse-compression/output latency”, ensuring deterministic end-to-end latency that meets real-time output requirements.

The goal of phase calibration is to estimate, at each subband frequency, the random phase term $\phi_{i}$ introduced by fast lock and to compensate subsequent echoes via online phase rotation. The system can use a calibration loop with fixed or non-fixed delay (denoted by $\tau_{c}$), and transmits two reference signals within the calibration slot to jointly estimate the loop delay and random phase term, reducing dependence on prior parameters. The two reference signals can be written as:

(17) $$x_{1}(t)=exp(j0t)$$

(18) $$x_{2}(t)=exp(j{2\pi f}_{a}t)$$

where $f_{a}$ denotes the frequency of the measurement signal. After passing through the same calibration loop, the complex baseband signals obtained at the i-th LO frequency $f_{c}(i)$ are:

(19) $$y_{1}(t)=x_{1}(t-\tau_{c})exp(-j{(2\pi f}_{c}(i)\tau_{c}{-\phi }_{i}))$$

(20) $$y_{2}(t)=x_{2}(t-\tau_{c})exp(-j{(2\pi f}_{c}(i)\tau_{c}-\phi_{i}))$$

It can be seen that $\tau_{c}$ and $\phi_{i}$ jointly determine the phases of the two calibration echoes. To remove the modulation term of the reference signal itself, complex correlation is performed between the baseband echo and the corresponding reference signal, yielding a modulation-independent phase term:

(21) $$\phi_{1}=2\pi f_{c}(i)\tau_{c}-\phi_{i}$$

(22) $$\phi_{2}=2\pi (f_{c}(i){+f_{a})\tau }_{c}-\phi_{i}$$

Thus, the parameters required for phase calibration can be solved. The equivalent loop-delay estimate is constructed from the phase difference of the two signals:

(23) $$\tau_{c}={(\phi }_{1}-\phi_{2})/2\pi f_{a}$$

and the random phase term $\phi_{i}$ is then obtained as:

(24) $${\phi_{i}=(\phi }_{1}+\phi_{2}+4{\pi f}_{c}(i)\tau_{c}+{2\pi f}_{a}\tau_{c})/2$$

The above calibration is executed immediately after fast lock, and the calibration results $\phi_{i}$ for each subband are stored before measurement starts. For each digital signal sequence output by the analog-to-digital converter (ADC), the random initial LO phase is compensated by complex multiplication to achieve phase alignment:

(25) $$z_{i}^{\prime }[n]=z_{i}[n]exp(-j\phi_{i}),n=0,1,\ldots ,L-1$$

where $z_{i}[n]$ is the discrete complex baseband echo sequence sampled in the measurement window for the i-th subband, $\phi_{i}$ is the phase-compensation term obtained and stored during the time-interleaved calibration, and L is the number of samples processed for that subband each time. This complex multiplication is equivalent to phase-aligning the sequence and can remove the random initial LO phase introduced by fast lock, maintaining coherent consistency during frequency-domain stitching and effectively suppressing sidelobe rise after synthesis. Furthermore, if the loop delay is known and constant, the calibration $\phi_{i}$ can be simplified to a single reference signal using (21), further shortening the calibration slot and reducing implementation complexity.

#### 2.2.2. Pulse Repetition and Coherent Accumulation

In real-time wideband synthesis, a direct engineering trade-off arises for a single subband measurement: on the one hand, reliable backscatter estimation and a clear range profile require sufficient SNR for each echo; on the other hand, improving per-echo SNR often implies a longer effective measurement time or a longer sampling window, resulting in more samples. More samples directly increase the throughput demand of online FFT, spectral shifting, and on-chip buffer read/write, reducing real-time margin. Therefore, “as few samples as possible for real time” and “as high SNR as possible for measurement quality” are core constraints that must be satisfied simultaneously.

To improve the equivalent SNR without significantly increasing the number of samples per processing instance, this paper adopts a subband pulse-repetition and coherent-accumulation strategy: at the same subband frequency, pulses are transmitted M times repeatedly, and under valid phase alignment the multiple echoes are coherently summed, so that the signal adds linearly while noise is relatively suppressed, achieving higher effective processing gain with a small per-pulse sample count. Let the baseband echo corresponding to the m-th transmission be:

(26) $$r_{m}(t)=\alpha s\left(t-\tau_{0}\right)exp(j\phi_{m})+n(t)$$

where m=1,2,…,M,s(t) is the reference transmitted signal, α is the target complex scattering coefficient, $\tau_{0}$ is the target delay, $\phi_{m}$ is the residual phase term of the m-th pulse, n(t) and is complex Gaussian noise.

For a stationary target, ideally all pulse echoes share a common delay $\tau_{0}$. If inter-pulse random LO phase or link phase drift exists, it can be estimated and compensated by the real-time calibration above, so that $\phi_{m}\approx 0$; after calibration, coherent summation yields the accumulated echo:

(27) $$r_{coh}(t)=\sum_{m=1}^{M}r_{m}(t)\approx M\alpha s\left(t-\tau_{0}\right)+n_{coh}(t)$$

According to the properties of coherent accumulation, under effective phase alignment, the signal-to-noise ratio of the accumulated echo ${SNR}_{coh}$ relative to that of a single baseband echo ${SNR}_{m}$, satisfies ${SNR}_{coh}=M{SNR}_{m}$.

**Figure 5:**
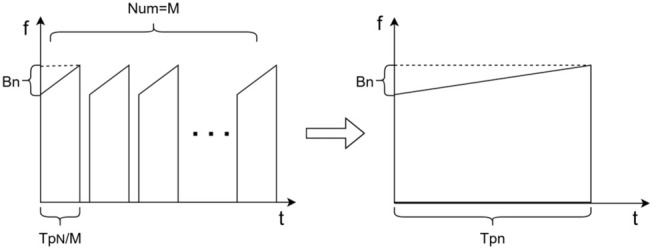
Energy illustration of subband pulse repetition and coherent accumulation.

As illustrated in Figure 5, this strategy can also be interpreted from an energy perspective: under the constraint of fixed total energy, if the pulse duration of each subband transmission is set to $T_{pN}/M$ and the pulse is repeated M times at the same frequency and coherently accumulated, the total energy is:

(28) $$E=\int {\left|s(t)\right|}^{2}dt={\left|A\right|}^{2}T_{pN}=M\cdot {\left|A\;\right|}^{2}\frac{T_{pN}}{M}$$

which is equivalent to the energy of a single transmission with duration $T_{pN}$. When phase alignment holds, the energy can be coherently recovered, improving effective measurement quality without increasing the FFT size per processing instance.

**Figure 6:**
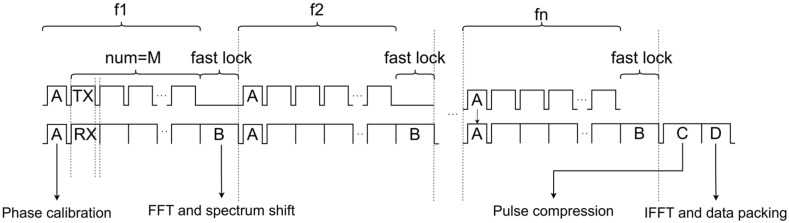
Schematic of timing after applying pulse repetition and coherent accumulation.

In addition, real-time bottlenecks often come not only from algorithmic computation but also from RF fast lock reconfiguration and settling time. If fast lock is too frequent, reconfiguration time will squeeze the effective measurement time, reducing the number of available echoes per unit time. As shown in Figure 6, this paper adopts a “grouped subband dwell” transmission schedule: For each subband i=1,2,3,…,N, after a single fast-lock configuration, the system performs M consecutive measurements and the corresponding processing at that frequency before hopping to the next subband.

With this strategy, a single wideband synthesis requires only N  frequency hops. The fast-lock overhead is amortized over M repeated acquisitions, resulting in an improved effective measurement duty cycle. It also provides a more stable pipeline window for FFT, spectral shifting, and stitching on the FPGA, which is beneficial for deterministic real-time output.

## 3. System Implementation

This section describes, from an engineering implementation perspective, how the proposed real-time “frequency-domain synthesis first, then pulse compression” method is realized on a low-cost SDR platform. Compared with the superheterodyne architecture, this work adopts a zero-intermediate-frequency (zero-IF) SDR platform, which provides a more cost-effective solution. The most typical and widely used implementation is the AD9361 SDR platform. The prototype system is built on a hardware platform consisting of an AD9361-based SDR RF front end and a Zynq-7020 SoC. Despite its low-cost advantage, this architecture still maintains a clear hardware–software partition and flexible configurability, thereby offering high system reconfigurability. The key goal is to perform online phase calibration, frequency-domain alignment/stitching, and unified pulse compression for multi-subband echoes under the instantaneous-bandwidth limitation of the AD9361, and to output range results with deterministic latency. The implementation is constrained by two factors: (1) AD9361 frequency fast lock involves non-negligible reconfiguration and settling time; and (2) the on-chip block random-access memory (BRAM) and digital signal processing (DSP) resources of the Zynq-7020 are limited, requiring a streaming, pipelined processing chain with minimal intermediate buffering. Therefore, we adopt a cooperative architecture of “closed-loop real-time processing in programmable logic (PL) + configuration and transmission in PS”, and organize the timing such that RF fast lock configuration overlaps with digital processing, making end-to-end latency predictable and budgetable.

### 3.1. System Framework and Task Partitioning

**Figure 7:**
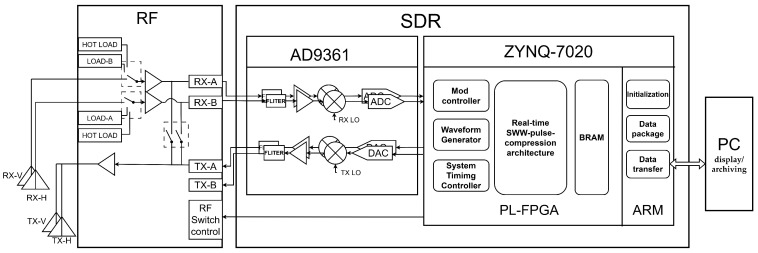
Overall radar system architecture based on the SDR platform.

**Figure 8:**
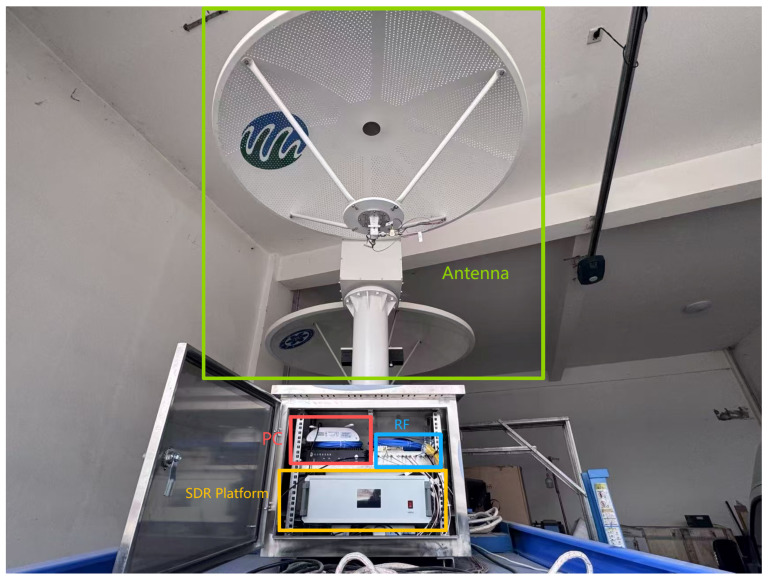
Photograph of the SDR-based ground scatterometer system. As labeled in the figure, the green box denotes the transmitting and receiving RF antennas, corresponding to the TX and RX terminals in Figure 7. The yellow box denotes the SDR platform, corresponding to the SDR module in Figure 7. The blue box denotes the RF front-end section, corresponding to the RF module in Figure 7. The red box denotes the host PC, corresponding to the PC unit in Figure 7.

The system follows an overall framework of “RF front end + SDR transceiver chip (AD9361) + Zynq-7020 digital processing + host display/archiving” (Figure 7). The corresponding photograph of the implemented system is shown in Figure 8.

The RF front end performs low-noise amplification and T/R switching and also integrates switching for the calibration path and receive-source selection, enabling switching between scatterometer measurement and calibration states. The calibration path not only supports phase calibration but also provides an internal calibration loop to compensate for system drift caused by temperature variation, mechanical vibration, and other disturbances. On the host side, the PC is responsible in the early stage for system-level Matlab R2018b simulation, LabVIEW 2013 software development, hardware development using Vivado 2018.3, and embedded development using SDK 2018.3, and in the later stage for data storage, processing, and display. The receive sources include a matched load and a hot load, which support radiometer-mode calibration; since this paper focuses on the scatterometer chain and the real-time processing architecture, details of the radiometer calibration are not discussed further. The AD9361 performs RF up/down conversion, I/Q processing, and analog-to-digital conversion/digital-to-analog converter (ADC/DAC), and provides a common sampling clock to the PL to ensure timing consistency between sampling and processing. The PL of the Zynq-7020 handles the real-time critical-path tasks, including waveform triggering and timing control, echo buffering, time-interleaved phase calibration, coherent accumulation of repeated pulses, zero-padded FFT, spectral shifting and stitching, frequency-domain matched filtering, IFFT, and writing the range profile to BRAM. The PS handles necessary but non-real-time management tasks, including system initialization, AD9361 parameter configuration, offline parameter downloading, result readout/packing, and Transmission Control Protocol (TCP) transmission to the host. The partition follows one principle: any computation and buffering that affect end-to-end real time are executed as deterministic pipelines in the PL, while the PS performs only configuration and data movement and is kept out of the real-time critical path.

The above results, together with Table 1, indicate that, under the BRAM/DSP constraints of a low-cost platform, implementing the proposed online synthesis and pulse-compression chain is feasible. Table 2 lists the system design parameters, including hardware parameters as well as antenna, frequency, bandwidth, and sensitivity. The system is designed for a dual-band (L and C) ground-based scatterometer; parameters that are common to both bands are described only once.

### 3.2. Deterministic Latency Budgeting

With fixed finite-state machine (FSM) scheduling and a fully pipelined data path, the end-to-end latency can be budgeted precisely in clock cycles. For subbands, the minimum processing time can be decomposed into two parts: (1) subband-dependent terms, including one calibration slot, one measurement window, and subsequent FFT and spectral shifting for each subband; and (2) fixed terms, including unified pulse compression after all subbands are stitched (frequency-domain complex multiplication + IFFT), data packing, and write-back to the output buffer. Table 3 provides detailed timing for each module.

Based on the PL-side cycle counts of each module (Table 3), the minimum processing latency can be written as:

(29) $$\begin{array}{c} T_{min}=291.84\cdot N+110.26\;\mu s \end{array}$$

where 291.84·N reflects the per-subband budget for calibration/measurement/FFT shifting, and 110.26 μs is the fixed overhead for unified pulse compression and output packing. In this system, N=5; thus, the minimum processing latency is approximately 1.57 ms, where the receive-window time is about 1.2 ms and the total time for computation and result presentation is 0.37 ms. Since the critical path is determined by an FSM driven by a synchronous clock and fixed pipelines, the end-to-end latency is highly deterministic and repeatable, providing a timing basis for subsequent real-time closed-loop measurement and calibration experiments.

Compared with traditional non-real-time processing methods, each frequency-band switch typically requires at least 0.1 s, so completing one full cycle of five subbands takes no less than 0.5 s. In contrast, the proposed onboard processing method reduces the processing latency to the millisecond level. For a real-time implementation that adopts the order of pulse compression first and then spectral shifting, the BRAM utilization reaches 129.8/140 (92.71%), and the minimum latency is $T_{min}=310.47\cdot N+70.26\;\mu s=1.62\;ms$. By comparison, the proposed method improves the time efficiency by 3.18% while reducing the BRAM pressure by 13.07%. This reduction in BRAM usage is important because it leaves sufficient resource margin for inserting an Integrated Logic Analyzer (ILA) for hardware debugging.

## 4. Experiments and Results

### 4.1. Cable Loopback Experiment: Range Resolution and Real-Time Verification

**Figure 9:**
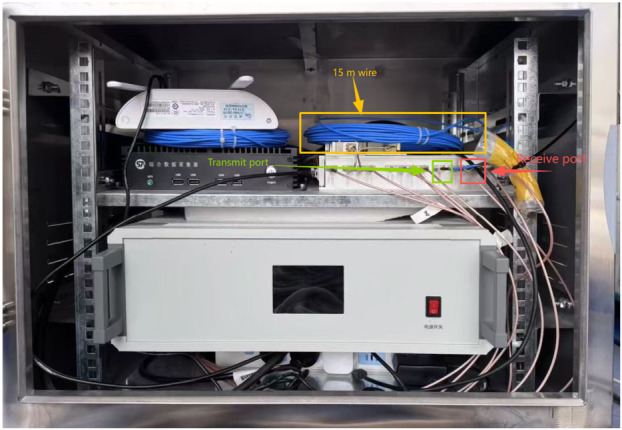
Photograph of the loopback experiment. A cable with a one-way length of 15 m and an attenuation of 30 dB was employed in the experiment. The system signal was transmitted from the transmitting port, propagated through the cable to the receiving port, and was then processed digitally to obtain the measurement result.

To verify the correctness and system-level performance of the proposed real-time chain of “frequency-domain synthesis first, then pulse compression”, a cable loopback experiment with a delay line was conducted. The field photograph of the loopback experiment is shown in Figure 9.

The delay line connects the transmitter and receiver to form an equivalent point-target echo. For each subband, online phase calibration is performed after fast lock and the compensation parameters are written; the echo is then acquired and processed onboard by frequency-domain alignment/stitching and unified pulse compression, outputting range-compressed results for point-target response (PTR) evaluation.

**Figure 10:**
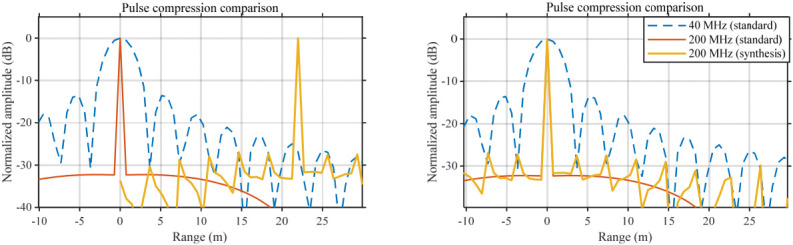
Loopback pulse-compression results of the 200 MHz multi-subband synthesis and the shifted plot. (Blue dashed line: ideal 40 MHz single-subband response; red solid line: 200 MHz single-subband response.)

To enable fair comparisons across different modes and bandwidths, the PTR curves are normalized by the main peak. Figure 10 compares the normalized range response for five-subband synthesis (200 MHz) with the ideal responses of a 40 MHz single band mode and a 200 MHz single band mode; the five-subband synthesis result is additionally shifted for clearer visualization.

From (16), the range resolution is determined by the equivalent bandwidth. When B = 200 MHz, the theoretical range resolution is ρ = 0.75 m. The experimental results show that the mainlobe is clearly wider in the 40 MHz single-subband mode, whereas under the equivalent 200 MHz bandwidth formed by multi-subband frequency-domain stitching, the mainlobe becomes significantly narrower and is almost identical to the standard 200 MHz reference result. These results indicate that, after coherent synthesis of multiple subbands, the range resolution corresponding to the equivalent bandwidth can nearly reach that of the standard 200 MHz wideband case. Hence, (16) is experimentally validated, and the results further confirm that multi-subband synthesis effectively expands the equivalent system bandwidth and delivers the expected range-resolution enhancement. Accordingly, the correctness of the onboard real-time synthesis-and-pulse-compression chain is also verified.

For sidelobe suppression, the PSLR and the ISLR are computed.

Table 4 compares the standard 200 MHz reference result with the proposed 200 MHz synthesis result. The proposed method achieves PSLR = −27.43 dB and ISLR = −12.38 dB, which are degraded relative to the reference (−32.25 dB and −15.90 dB). This degradation mainly comes from residual inter-subband amplitude/phase mismatch, guard bands, and stitching-boundary effects. This indicates that, although sidelobe degradation is introduced while obtaining the equivalent wideband response, the sidelobe level remains within the engineering tolerance acceptable for ground-based scatterometer measurements. Therefore, the proposed real-time multi-subband frequency-domain synthesis and pulse-compression method achieves a practical trade-off between resolution improvement and sidelobe controllability on a low-cost platform.

During the loopback measurements, a free-running hardware counter was used on the PL side to collect per-frame processing-cycle statistics, verifying the cycle determinism of the proposed onboard real-time chain.

**Figure 11:**
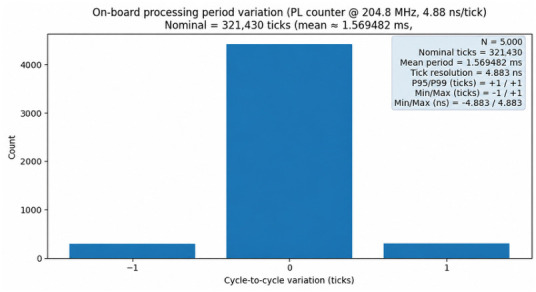
Distribution of onboard processing cycles per frame (5000 repetitions).

Together with the frame-period budget in Section 3 (about 1.57 ms), these hardware statistics demonstrate deterministic real-time output capability: under fixed clock and parameter settings, the per-frame processing period is predictable with a controllable upper bound and can stably support a continuous update rate of about 1/1.57 ms. This provides timing assurance for subsequent online computation and real-time display of backscatter coefficients and range profiles (Figure 11).

### 4.2. Field Measurements

**Figure 12:**
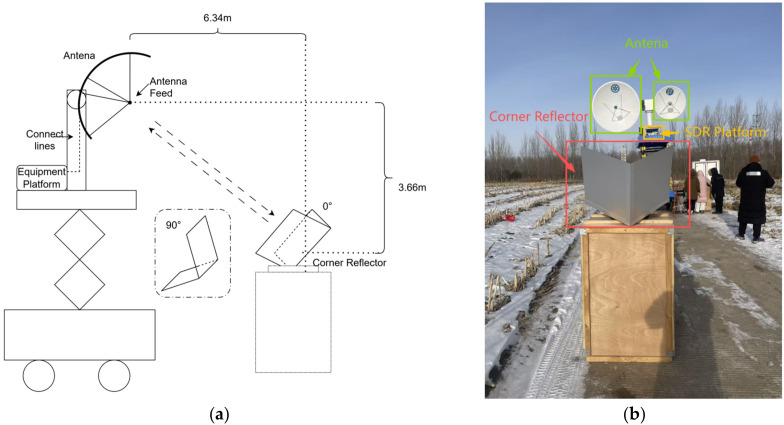

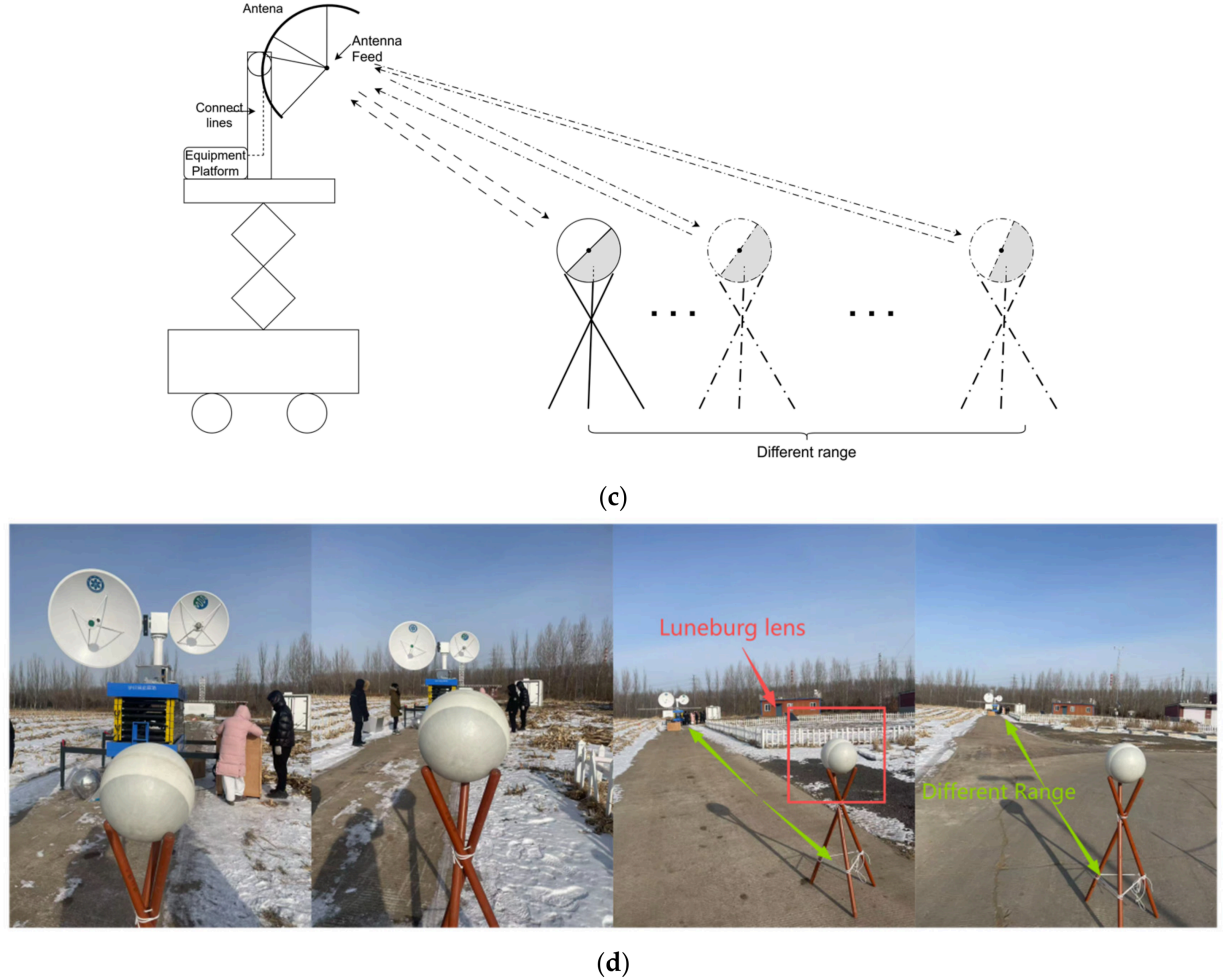
Photographs and schematics of the measurement setups. (**a**) Schematic of the polarimetric matrix measurement setup. (**b**) illustrates the polarization calibration setup using corner reflectors. (**c**) Schematic of target echo measurement at different ranges. This panel illustrates the measurement procedure for target echo power. Because the Luneburg lens has stable scattering characteristics, a well-defined echo response, and produces almost no cross-polarized component, it can effectively avoid the interference of cross-polarization in echo power verification and is therefore more suitable for examining the consistency between the measured echo power and the theoretical attenuation law [25]. For this reason, the Luneburg lens was selected as the reference target for point-target echo power measurements, and (**d**) shows the measurement configuration employing a Luneburg lens to acquire point-target echoes at different ranges (7.35 m, 8.10 m, 22.05 m, 38.96 m, 64.68 m, 84.53 m).

**Figure 13:**
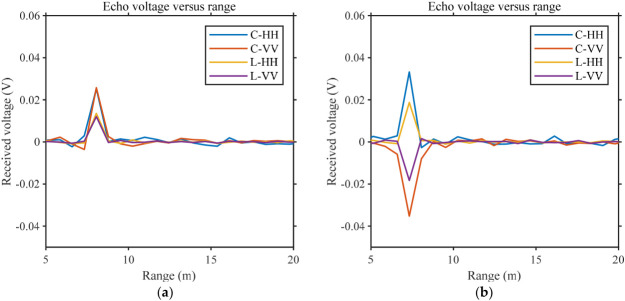
Measured echo voltages of the dihedral corner reflector at different orientation angles. The legend denotes the measured results for C-band HH polarization, C-band VV polarization, L-band HH polarization, and L-band VV polarization. (**a**) corresponds to the dihedral reflector oriented at 0°, for which the measured scattering matrix can be approximately expressed as $\left(\begin{array}{cc} 1 & 0\\ 0 & 1 \end{array}\right)$, while (**b**) corresponds to the dihedral reflector oriented at 90°, with an approximate scattering matrix given by $\left(\begin{array}{cc} 1 & 0\\ 0 & -1 \end{array}\right)$.

To verify the applicability of the proposed scheme in practical scattering measurements, polarimetric calibration was first carried out for the system. Dihedral and trihedral corner reflectors were used to examine the amplitude and phase consistency of the four polarization channels. The schematic of the measurement scenario is shown in Figure 12a. The dihedral corner reflector was positioned such that its center was located at a horizontal distance of 6.34 m and a vertical distance of 3.66 m from the antenna feed center. The placement was determined using an infrared rangefinder, and laser alignment was further employed to ensure that the centerline of the dihedral corner reflector was aligned with the antenna feed center. The field deployment is shown in Figure 12b. By changing the angle between the ridge line of the dihedral corner reflector and the normal direction, the polarimetric measurement relationship was established in conjunction with the scattering matrix of the reference target. Figure 13 presents a set of comparative results for the measured polarimetric scattering matrices. To more clearly demonstrate the polarimetric measurement performance, only the co-polarized channels are shown.

It can be observed that, at a fixed measurement range, when the dihedral corner reflector is placed at specific orientation angles, the HH and VV polarization channels exhibit stable and physically expected polarization-dependent responses. As the orientation angle of the dihedral reflector changes, the echo characteristics of each polarimetric channel vary accordingly, reflecting the response of the scattering matrix to changes in geometric orientation. These results indicate that the measured polarimetric echoes are consistent with the theoretical scattering behavior of the dihedral reflector under the corresponding orientations, thereby confirming the correctness and stability of the established polarimetric measurement relationship. This demonstrates that the proposed measurement system possesses reliable polarimetric discrimination capability, providing effective support for subsequent polarimetric measurements and echo consistency verification.

**Figure 14:**
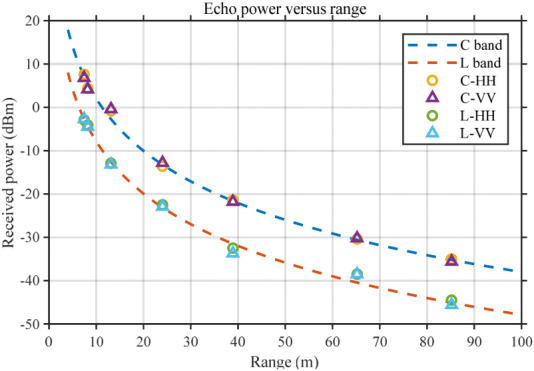
Corresponding results. The figure presents the measured echo power at different ranges, where the legend denotes measurements for C-band HH polarization, C-band VV polarization, L-band HH polarization, and L-band VV polarization, respectively, thereby illustrating the correspondence between the measured echo power and the theoretical values at different ranges.

Subsequently, outdoor experiments were carried out. As illustrated in Figure 12c, a reference target with stable scattering characteristics, such as a Luneburg lens, was placed at different ranges. During the field measurements, the antenna was pointed toward the reference target and aligned using a laser pointer to ensure reliable echo acquisition. This arrangement enabled the measurement of point-target echo power at different distances, and the corresponding field deployment is shown in Figure 12d. The corresponding results are shown in Figure 14. Since a Luneburg lens produces negligible cross-polarized scattering, cross-polarized channel results are not presented.

The blue solid curves (C-band) and orange dashed curves (L-band) represent the fitted power responses based on the radar equation,

(30) $$P_{r}(R)=\frac{P_{t}G^{2}\lambda^{2}\sigma }{(4\pi {)}^{3}R^{4}L}$$

where $P_{t}$ denotes the transmit power, G denotes the antenna gain, λ denotes the wavelength, σ denotes the known radar cross section (RCS) of the calibration target at the current band, R denotes the range, $P_{r}$ denotes the echo power, and L denotes the system-loss factor.

It can be observed that the HH and VV polarization channels at both frequency bands exhibit similar behavior: the measured echo power decreases monotonically with increasing range. The measured data points do not lie exactly on a single fitted curve; they fluctuate slightly above and below the fitted response. In both near-range and mid-range regions, some data points are marginally higher or lower than the fitted curve; however, the overall dispersion remains bounded and shows no systematic bias. Such random fluctuations around the fitted curve indicate that the measured power variation follows the theoretical attenuation law, while also reflecting unavoidable scattering fluctuations and measurement uncertainties under field conditions.

The above observations suggest that the proposed system captures the expected range-dependent attenuation while exhibiting realistic measurement dispersion in field environments. The main causes of the fluctuations include: amplitude variations of the target RCS due to small changes in attitude and alignment, constructive/destructive interference from environmental multipath, as well as noise-floor variation and slight gain drift of the system. In addition, finite range resolution can noticeably affect power extraction within a range gate: when the echo mainlobe width is comparable to the gate width, energy can leak into adjacent range bins via mainlobe leakage and sidelobe coupling, causing the integrated power in some gates to be slightly higher or lower than the fitted value. Therefore, the observed dispersion is consistent with practical field measurements and with the system’s range-resolution capability.

**Figure 15:**
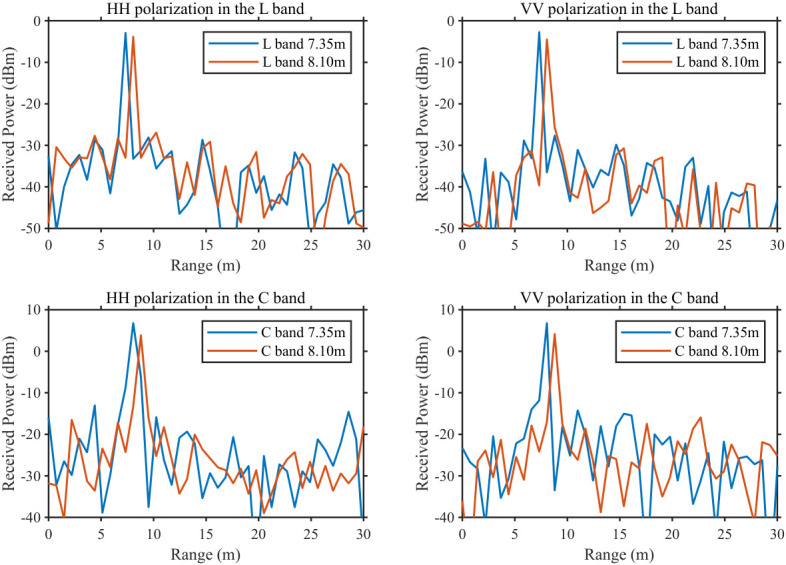
Point-target responses at 7.35 m and 8.10 m.

For comparison of adjacent point-target responses, Figure 15 shows the echo-power curves at 7.35 m and 8.10 m for both L and C bands. The range separation is 0.75 m, consistent with the theoretical range resolution of the 200 MHz equivalent bandwidth. The separation exceeds the 3 dB mainlobe width and does not fall within the 6 dB mainlobe range; thus, two distinct and separable response peaks are observed without mainlobe overlap or unresolved aliasing, experimentally verifying sub-meter range resolution.

The sidelobe metrics for the four cases are: PSLR = −22.80 dB, −23.23 dB, −20.06 dB, and −21.04 dB; corresponding ISLR = −11.38 dB, −12.12 dB, −9.38 dB, and −8.60 dB. The differences from the ideal case mainly arise from field noise, link loss, and non-ideal factors such as multipath and clutter. Overall, the sidelobe levels and peak separability still demonstrate that the real-time wideband synthesis and pulse-compression chain operates stably and accurately in the system.

**Figure 16:**
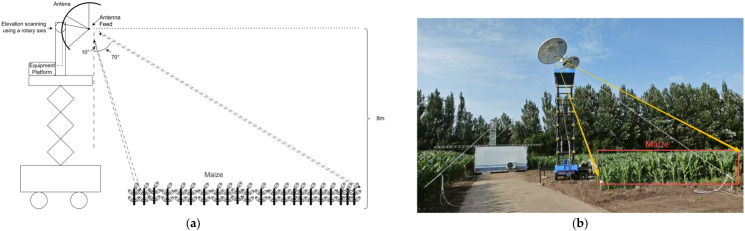
Schematic and field photograph of farmland scattering measurements using the vehicle-mounted ground-based scatterometer. (**a**) Schematic of the farmland scattering measurement. To better capture the statistical characteristics of the crop canopy, the antenna height should be higher than 5.6 m. The maximum antenna height of the vehicle-mounted platform is 16 m. In this experiment, the antenna height was fixed at 8 m, and the azimuth angle was kept constant. Under this condition, the antenna scanned the incidence angle range from 10° to 70° through the pitching axis. (**b**) Field photograph of the farmland scattering experiment.

Subsequently, a vehicle-mounted ground-based scatterometer experiment over farmland was conducted, as shown in Figure 16.

**Figure 17:**
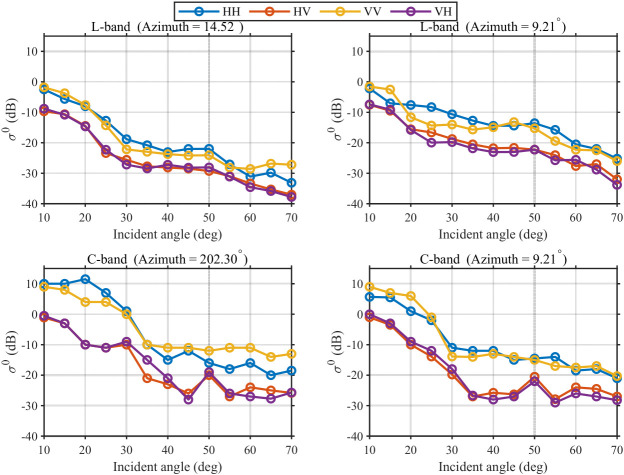
L-band and C-band backscatter coefficient versus incidence angle at different azimuth angles.

Field backscatter measurements were conducted with the antenna pointing toward the maize crop area. The antenna height was kept constant; the platform azimuth angle was adjusted; for each azimuth, an elevation scan was performed and experimental parameters were recorded. Echoes of each polarization were collected, and the backscatter coefficient was computed to obtain the curves. Neglecting blockage effects of the vehicle platform, measurements for incidence angles from 10° to 70° were summarized, as shown in Figure 17.

From the results at different azimuth angles, the overall trends of the backscatter coefficient versus incidence angle are highly consistent across polarization channels: over approximately 10–70°, the backscatter coefficients decrease with increasing incidence angle, with cross-polarization consistently lower than co-polarization. The decline is rapid in the quasi-specular region, moderate in the plateau region, and becomes steeper with stronger fluctuations in the interference region, consistent with the typical angular response of backscatter models [26,27].

Although the same area was measured, the polarization channels show observable amplitude differences and local shape variations across azimuth angles, indicating that changing azimuth alters the scattering geometry and the projection of the effective roughness spectrum, thereby affecting backscatter. Meanwhile, the differences are not random drift but scale with the azimuth change: when the azimuth difference is small (e.g., 9.21° versus 14.52°), the curves remain highly consistent in overall shape and turning points, with only small offsets, reflecting a physically reasonable azimuth sensitivity. Compared with L-band, C-band exhibits more pronounced local undulations at mid-to-large incidence angles, indicating higher sensitivity to fine-scale structure and roughness spectra.

The slight rebound or fluctuation around 60–70° may be attributed to soil–canopy interaction, local structural heterogeneity, or localized scattering enhancement associated with Bragg-type resonance effects [28], which are commonly explainable phenomena in field measurements.

Furthermore, for the same azimuth angle of 9.21°, L-band and C-band measurements of the same target show consistent incidence-angle response shapes, main transition regions, and relative polarization relationships. The differences are mainly in magnitude and local undulations due to different wavelength sensitivities to scattering mechanisms at different spatial scales. This cross-band consistency further supports the accuracy and reliability of the measurements.

**Figure 18:**
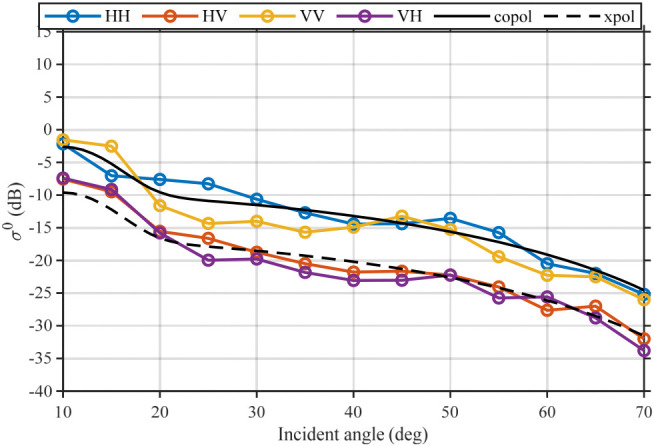
Compares the measured curves with the model predictions, where the model curves for co-polarization and cross-polarization are plotted as a black solid line (co-polarization) and a black dashed line (cross-polarization), respectively.

To further assess whether the measured angular trends are physically consistent, we compare the measurements with a Tor Vergata (TVG) scattering model [29,30] under locally constrained crop parameters. By applying localized constraints on crop-related parameters (including Soil moisture\Leaf area index\Canopy water content\Plant height\Canopy temperature\Maize stalk diameter\Maize stalk height\Biomass\Soil temperature\Sand volume fraction\Clay volume fraction\Soil bulk density\Soil roughness root mean square height\Soil roughness correlation length), the simulated $\sigma_{0}$–incidence-angle curves were obtained, as shown in Figure 18 (taking a representative azimuth angle as an example).

It can be observed that the model curves show good agreement with the measurements in terms of overall trend and magnitude range: the measured curves mostly fluctuate around the model-predicted copol/xpol trajectories and exhibit consistent angular dependence. This indicates that, under localized parameter constraints, the TVG model can reasonably characterize the angular response of the crop scene, thereby further validating the reliability of the measured results and the engineering feasibility of the proposed measurement system from a model-consistency perspective. The local deviations and fluctuations of the measurements relative to the model curves may result from spatial heterogeneity within the field, row-orientation effects, mixed contributions of multiple scattering mechanisms, as well as measurement noise and calibration uncertainties.

Overall, the cross-azimuth consistency shown in Figure 17 and the model comparison in Figure 18 jointly demonstrate that the low-cost vehicle-mounted SDR scatterometer can stably capture the $\sigma_{0}$–incidence-angle dependence and provides repeatable observations of environmental sensitivity features, thereby supporting the feasibility of the proposed real-time multi-subband frequency-domain synthesis and pulse-compression architecture.

## 5. Conclusions

This paper proposes an onboard real-time processing architecture for a low-cost vehicle-mounted SDR scatterometer, achieving equivalent wideband operation via coherent multi-subband synthesis. By performing frequency-domain subband synthesis before pulse compression, the pipeline reduces repeated computation and naturally fits streaming FPGA implementation. The slot-based online phase calibration compensates for the random LO phase introduced by fast lock, enabling coherent stitching. The within-subband pulse repetition provides coherent processing gain without increasing the per-frame FFT size. An implementation based on Zynq-7020 and AD9361 achieves a nominal 1.57 ms frame period with deterministic real-time output and ±1 tick jitter. The loopback experiment verifies the resolution improvement brought by 200 MHz synthesis and reports sidelobe metrics (PSLR −27.43 dB, ISLR −12.38 dB).

Field experiments further show that the system output is stable and repeatable under outdoor conditions: for incidence-angle scans at different azimuth angles, the $\sigma_{0}$ trends of all polarization channels agree with classical angular response behavior, and the azimuth-induced amplitude differences are consistent with scattering geometry rather than random drift. Under the same azimuth angle, L/C-band measurements of the same target preserve consistent curve shapes, further validating the effectiveness and engineering practicality of the measurement chain.

The main limitations are as follows: the number of subbands and inter-subband amplitude/phase consistency limit the attainable equivalent bandwidth and the upper bound of range-resolution improvement; long-range measurements are constrained by transmit power, antenna gain, and receiver dynamic range, making echoes more susceptible to noise and clutter; and the real-time chain is sensitive to sampling-rate and sample-count settings, so platforms with different specifications require re-parameter planning. Future work will focus on improving inter-subband amplitude equalization, optimizing overlap and guard-band design, enhancing online calibration, and parameterizing the frame structure and FFT size to improve portability and real-time margin.

## Figures and Tables

**Table 1:** Hardware platform parameters and resource utilization.

Parameters Name	Value
System Clock	204.8 MHz
BRAM	111.5/140 (79.64%)
DSP	24.55%
Fs	51.2 MHz (I/Q)
Tp	5 μs
Tr (time of receive)	5.9965 μs
Bn	40 MHz
N	5

**Table 2:** System parameters.

Parameters Name	Value
Range of Frequency	1.3 GHz~1.5 GHz (L) 5.5 GHz~5.7 GHz (C)
Distance measured	149.30 m
Range resolution	≈0.75 m
Receiver sensitivity	≤−100 dBm
Antenna polarization	VV, HH, VH, HV
Transmit power	≥25 dBm
Modulation waveform	LFM (linear frequency-modulated)
Receiver dynamic range	≥55 dB
Antenna 3-dB beamwidth	3.8° (L) 1.5° (C)
Antenna peak gain	27 dB (L) 35 dB (C)

**Table 3:** Timing budget of each processing module under a 204.8 MHz clock.

MOD	The Number of Clock Cycles	The Number in Every f(i)	Total Time (μs)
TX	1024	40	200
RX	1228	40	239.84
Phase calibration	1024	1	5
FFT and spectrum shift	5249	1	25.63
Pulse compression	8192	1	40
fast lock	9626	1	47
IFFT and data packing	14,388	1	70.26
TOTAL			291.84·N+110.26

**Table 4:** Comparison of PSLR and ISLR between standard and synthesis results.

Signal	PSLR (dB)	ISLR (dB)
200 MHz Standard	−32.25	−15.9
200 MHz Synthesis	−27.43	−12.38

## Data Availability

Data are contained within the article.
